# Time to address gender discrimination and inequality in the health workforce

**DOI:** 10.1186/1478-4491-12-25

**Published:** 2014-05-06

**Authors:** Constance Newman

**Affiliations:** 1 IntraHealth International, 6340 Quadrangle Drive, Suite 200, Chapel Hill, NC 27517, USA

**Keywords:** Gender, Bias, Discrimination, Stereotyping, Occupational segregation, HRH, Health workforce, Labour rights

## Abstract

Gender is a key factor operating in the health workforce. Recent research evidence points to systemic gender discrimination and inequalities in health pre-service and in-service education and employment systems. Human resources for health (HRH) leaders’ and researchers’ lack of concerted attention to these inequalities is striking, given the recognition of other forms of discrimination in international labour rights and employment law discourse. If not acted upon, gender discrimination and inequalities result in systems inefficiencies that impede the development of the robust workforces needed to respond to today’s critical health care needs.

This commentary makes the case that there is a clear need for sex- and age-disaggregated and qualitative data to more precisely illuminate gender-related trends and dynamics in the health workforce. Because of their importance for measurement, the paper also presents definitions and examples of sex or gender discrimination and offers specific case examples.

At a broader level, the commentary argues that gender equality should be an HRH research, leadership, and governance priority, where the aim is to strengthen health pre-service and continuing professional education and employment systems to achieve better health systems outcomes, including better health coverage. Good HRH leadership, governance, and management involve recognizing the diversity of health workforces, acknowledging gender constraints and opportunities, eliminating gender discrimination and equalizing opportunity, making health systems responsive to life course events, and protecting health workers’ labour rights at all levels. A number of global, national and institution-level actions are proposed to move the gender equality and HRH agendas forward.

## Introduction

HRH experts have noted that health workforce gender imbalances are a major challenge for health policy-makers [1]. They have also observed that improving gender equity is essential to strengthen workforce numbers, distribution, and skill mix and that HR policy and planning failures are traceable to HRH leaders’ failure to account for gender [2]. Global dialogue on the post-2015 agenda currently focuses on gender equality as a development goal in its own right [3,4], and this should be central to dialogue and debate related to health workforce shortages.

In this commentary, a case is presented for paying more attention to gender discrimination and inequality as they operate in the health workforce. In addition to reviewing gender in the HRH literature and describing the ways that gender has been framed, the commentary considers ways to define and think about gender inequality and discrimination in the workforce. It presents research evidence from Kenya, Uganda, and elsewhere to illustrate unequal opportunity and workplace gender discrimination, and suggests actions to move the gender equality and HRH agendas forward.

### Key concepts

Gender inequalities are systems inefficiencies that contribute to clogged health worker educational pipelines, recruitment bottlenecks, attrition, and worker maldistribution in formal and non-formal health workforces. Fostering gender equality increases the likelihood of women and men having an equal chance of choosing a health occupation, acquiring requisite skills and knowledge, being hired and fairly paid, and enjoying equal treatment and advancement opportunities. Promoting nondiscrimination and gender equality in policy and planning can, for example, target gender stereotypes that may keep men from entering female-identified jobs such as nursing and HIV/AIDS care and support [5]. Equality-focused policies can also recognize that female health workers juggle life cycle events such as childbirth and caregiving with career progression and promote measures to empower women to enter and remain in the health labour market.

Gender has been implicated in a wide variety of health workforce considerations. These include, for example:

• Workforce structures and concentration hierarchies [1,6,7]

• Client-provider interactions [8]

• The female composition of the workforce [9], particularly at the primary level

• The experiences of female nurses, community health workers, and home carers, including the unpaid, underpaid, unsupported, and disproportionately female workforces that often constitute the informal care economy [2,10]

• The ways in which (especially) female workers’ normal life experiences (for example, pregnancy, child care) become problematized due to their incompatibility with male work models that do not take life course events into account [11,12]

• Access to non-pecuniary rewards [13], continuing education, and professional training [14]

• Differences in wages [15,16]

• Disparities in workplace safety knowledge [17]

• Blindness of occupational health research [18]

• Health worker mobility [19]

• Perceptions of health and quality of life among health workers [20].

HRH experts describe gender inequality in multifaceted ways as it operates in and through the health workforce, encompassing constructs such as gender equity, equality, differentials, gaps, imbalance, parity, bias, skewness, and discrimination. There appears to be no unified, holistic conceptual understanding to frame significant gender inequalities as they operate in the health workforce and examine possible workforce and health systems consequences.

### Gender discrimination

Discrimination is a particularly important aspect of gender in the workforce [11,12,21,22]. Table 1 defines discrimination (as well as equal opportunity and nondiscrimination) and provides key related definitions for this section and the rest of the paper.

**Table 1:** Key definitions

	
**Gender discrimination**	Any distinction, exclusion, or restriction made on the basis of socially constructed gender roles and norms that prevents a person from enjoying full human rights [23].
**Discrimination in employment and occupation**	Practices that place individuals in a subordinate or disadvantaged position in the workplace or labour market because of characteristics (race, religion, sex, political opinion, national extraction, social origin, or other attribute) that bear no relation to the person’s competencies or the inherent requirements of the job [24].
**Basis for sex or gender discrimination**	Distinctions made on the basis of biological characteristics and functions that distinguish men and women (for example, height, weight) or on the basis of social differences between men and women (for example, marital status, family situation, maternity).^a^
**Bias**	An inclination or prejudice for or against one person or group, especially in a way considered to be unfair, that often results in discrimination [25].
**Equal opportunity and nondiscrimination**	The offering of employment, pay, or promotion to all, without discrimination as to sex, race, color, disability, and so forth [26].
**Gender equality in the workforce**	A condition where women and men can enter the health occupation of their choice, develop the requisite skills and knowledge, be fairly paid, enjoy fair and safe working conditions, and advance in a career, without reference to gender; implies that workplaces are structured to integrate family and work to reflect the value of caregiving for women and men [27].

^a^Women are most commonly affected by sex discrimination; however, prohibition of discrimination based on sex does not address all the types of inequalities women face in the workforce [28].

The empirical study of discrimination is challenging, due to differing perceptions, measurement approaches, and unwillingness to publicly acknowledge or report it [29-31]. Also, workers who might be willing to report discrimination may simply not have access to information substantiating it. The term ‘gender bias’ sometimes appears in HRH-related reviews instead of discrimination [7,13,18], and it is not clear if the term includes discrimination. Discrimination remains implicit in some research findings. For example, a recent study in Tanzania [32] refers to *gender skewness*, though a closer reading reveals that part of the skewness consists of occupational segregation, an enduring form of workforce inequality and discrimination [33,34]. A recent study on gender and wages [16] found that ‘An increase in the percentage of women in an occupation has a large downward effect on its wage rank’ (p. 1), with one additional percentage point of women in an occupation being associated with a decrease in the wage rank of 8% in standardized USD (p. 13). Although the authors do not explicitly address the link between the discrimination that is occupational segregation [33] and a gender wage gap, they conclude that ‘This result is in line with devaluation theory arguing that “female” tasks and skills are devaluated in the labour market’ ([16], p. 13). This echoes Marini’s 25-year-old finding that the more an occupation is female-identified, the lower are the wages for that occupation [35]. However, the more recent study cited here [16] is a milestone for the field, and future HRH research should continue to connect the evidentiary dots between occupational composition, segregation, and the gender wage gap. (This link is supported by other research, which frames it as discrimination [36]).

Gender and HRH experts have argued for more research and sex-disaggregated data to strengthen understanding of gender as it affects health workers, especially in developing countries [2,7,13,21]. The lack of high-quality data may be a reason for limited attention to gender discrimination on the part of HRH stakeholders [13]. Indeed, an important 24-country study [37], which included health policy-makers, researchers, and community and civil society representatives, demonstrated that current HRH research priorities do not explicitly include gender. This suggests that HRH leaders do not have enough information about the diversity of health workforces, the different life and work opportunities and constraints faced by health workers, the ways that some may be disadvantaged by these, the way that men and women are concentrated (that is, segregated) in particular health occupations [38] and at different hierarchical levels, and the consequences these factors may have for recruitment, productivity, and retention. In fact, the lack of concerted attention to gender discrimination in HRH research, policy, and practice is striking, given its recognition in other sectors such as employment-related jurisprudence and the protection of human (labour) rights [39-44]. This further suggests that HRH leaders do not frame inequalities between health workers in terms of human rights, and that the protections from discrimination in education, occupation, and employment offered through international or national policies and laws are not routinely extended to health workers.

Given the lack of specificity of terms, it is quite possible that HRH inattention to gender discrimination is due to a lack of clarity and consensus about what it is and how it manifests itself in the health workforce. If so, it is useful to begin with concrete ways in which to think about it. While not all things gender in the workforce are discrimination, some are, and for those, HRH leaders need common ways to define, frame, measure, recognize, discuss, and act on it. Table 2 presents common forms and types of workforce gender discrimination.

**Table 2:** Forms and types of sex or gender discrimination

	• Indirect: an apparently neutral situation, measure, law, criterion, policy, or practice that disproportionately and negatively affects persons from a particular group (for example, exclusion of domestic, informal, or home health workers from protective labour legislation)
**Gender discrimination can be direct [**[24]**] and overt [**[41]**] or indirect [**[24]**]:**	• Direct: intentional or explicit discrimination, in law or in practice (for example, job advertisement excluding women or men), arising when factors unrelated to merit, ability, or potential are used as an explicit reason for excluding or restricting participation of a person or group
	• Overt: hostility or a ‘discriminatory animus’ toward women in the workforce
	• Indirect: an apparently neutral situation, measure, law, criterion, policy, or practice that disproportionately and negatively affects persons from a particular group (for example, exclusion of domestic, informal, or home health workers from protective labour legislation)
**Sex or gender discrimination can take multiple forms:**	• Vertical and horizontal occupational gender segregation
	• Wage discrimination
	• Sexual harassment or unwanted or offensive conduct that creates an intimidating, hostile, or humiliating school or work environment
**Sex or gender discrimination can be based on a variety of factors:**	• Marital status or pregnancy
	• Family (or ‘caregiver’) responsibilities
	• Age
**Disparate treatment or impact [**[41]**] amounts to** ‘**second**-**class citizenship’:**	• Can occur at any phase of the employment relationship
	• Consists of intentional or unintentional restrictions or exclusions that have bias or discrimination as their source
	• Results in disadvantages in recruitment, hiring, compensation, promotion, or work conditions
**Gender stereotyping [**[41]**] can be involved in all forms of gender discrimination:**	• Expresses and reinforces women’s traditional - and inferior - role in the workforce
	• Can affect occupational or employment decisions (for example, recruitment, hiring, promotion, termination)

Key points from Table 2 are that gender discrimination can be direct or indirect, overt or covert, and associated with disparate and negative consequences for the person who experiences it. Further, women may experience multiple forms of discrimination in the workforce based on their biological sex and gender identity. Table 3 lists the negative effects of gender discrimination and inequality and the positive effects of equal opportunity and gender equality.

**Table 3:** Negative effects of gender discrimination and inequality and positive effects of equal opportunity and gender equality

**Negative effects**	**Positive effects**
• Entry into health occupations impeded	• Equal access to professional education, requisite skills, and knowledge
• Clogged health worker education pipeline	• Increased health worker pipeline
• Workers' career progression impeded	• Equal chance of being hired, fairly paid, and enjoying equal treatment and advancement opportunities
• Workers experience work/family conflict, low morale, stress, lower productivity	• Female health workers better able to juggle life events
• Recruitment bottlenecks	• Better work/life integration for all health workers, less stress
• Worker maldistribution	• Better morale and productivity
• Workplaces experience absenteeism, attrition	• Increased retention
• Limited pool of motivated health workers to deal with today’s health challenges	• More health workers
	• More health services

The next section presents evidence of gender discrimination and inequality in health pre-service and employment systems, respectively.

### Gender discrimination and inequality: selected evidence

#### *Pre-service systems*

A 2012 systematic literature review [45] of gender in health pre-service education and general tertiary systems in several countries identified 51 interventions to counter disadvantage stemming from pregnancy and family responsibilities and sexual harassment, as well as interventions promoting gender equality generally. The review recommended implementing a multilevel ‘basic bundle’ of interventions to target the roots of discrimination and violence, eliminate impunity for sexual harassment, and transform school and work arrangements so women are not disadvantaged by pregnancy and family caregiving.

#### *Employment systems*

A national-level multimethod study of Rwanda’s health employment system [11] (which resulted in revisions in national labour law) used focus groups, written surveys using random sampling, key informant interviews, and document reviews to identify factors affecting health workers’ experiences, work expectations, and career paths. Findings included evidence of negative stereotypes of female workers, sexual harassment, lack of support to mothers in workplaces, pregnancy-based and caregiver discrimination, and vertical segregation (the concentration of men in topmost management). These factors contributed to workplace violence.

Small-scale multimethod studies of gender discrimination and inequality in the public and private health employment systems in Zambia and Uganda (unpublished observations) found a lack of policy responsiveness to life course events for workers with family responsibilities, as well as evidence of sexual harassment, gender bias, and occupational gender segregation. The studies employed multiple focus group discussions over several geographical sites with separate group discussions involving male and female managers and health workers. These focus groups yielded evidence of pro-male bias and a ‘discriminatory animus’ [41] toward female managers, manifested in stereotypes of women’s emotionality, mood swings, tendency to make mistakes, lesser productivity, unreliability, disorganization, vengefulness, lesser mental agility, inability to handle power, weakness, indecisiveness, and incompetence. In the Rwanda study mentioned earlier [11], a study participant remarked, ‘*Women are not even capable of pulling out a tooth*’.

Together, the Rwanda, Uganda, and Zambia studies highlight the gender-related distinctions and restrictions associated with discrimination in employment systems. Indeed, they suggest a constellation of co-occurring biases or discriminatory actions requiring concerted HRH policy attention and management response. It is worth mentioning that the evidence from these three African countries is presented only because the countries had HRH projects supported by some research funding. This suggests that there is a vein of evidence ready to be mined in other countries and regions.

### Supplementing special studies with human resources information system data

As we have just seen, qualitative data generated by multimethod special studies can reveal some of the gender dynamics in the health workforce. These data can be supplemented by sex- and age- disaggregated administrative and human resources information systems (HRIS) data to explore gender trends in the workforce. Illustrative examples from Kenya and Uganda follow.

#### *Kenya*

**Figure 1:**
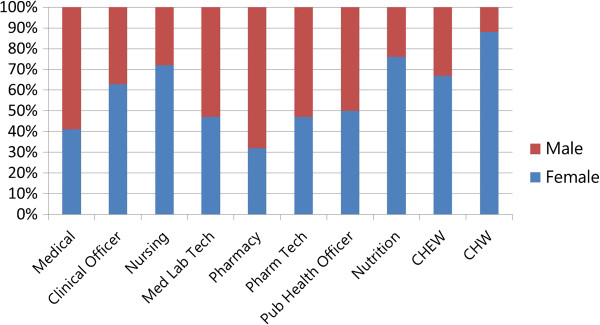
**Percentage of students by cadre training programme and sex, Kenya 2010 (N = 42 institutions).**

**Figure 2:**
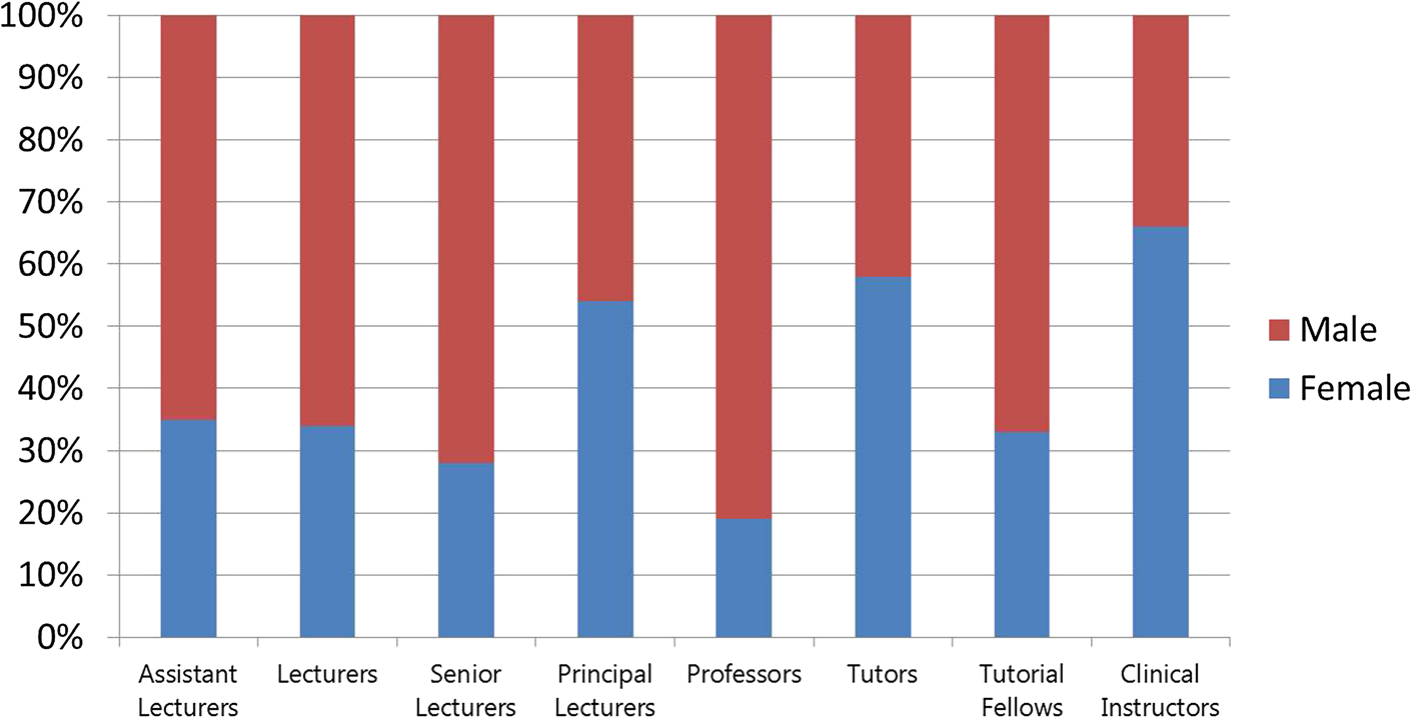
**Number of faculty by position and sex in 21 nursing-only education institutions, Kenya 2010.**

**Figure 3:**
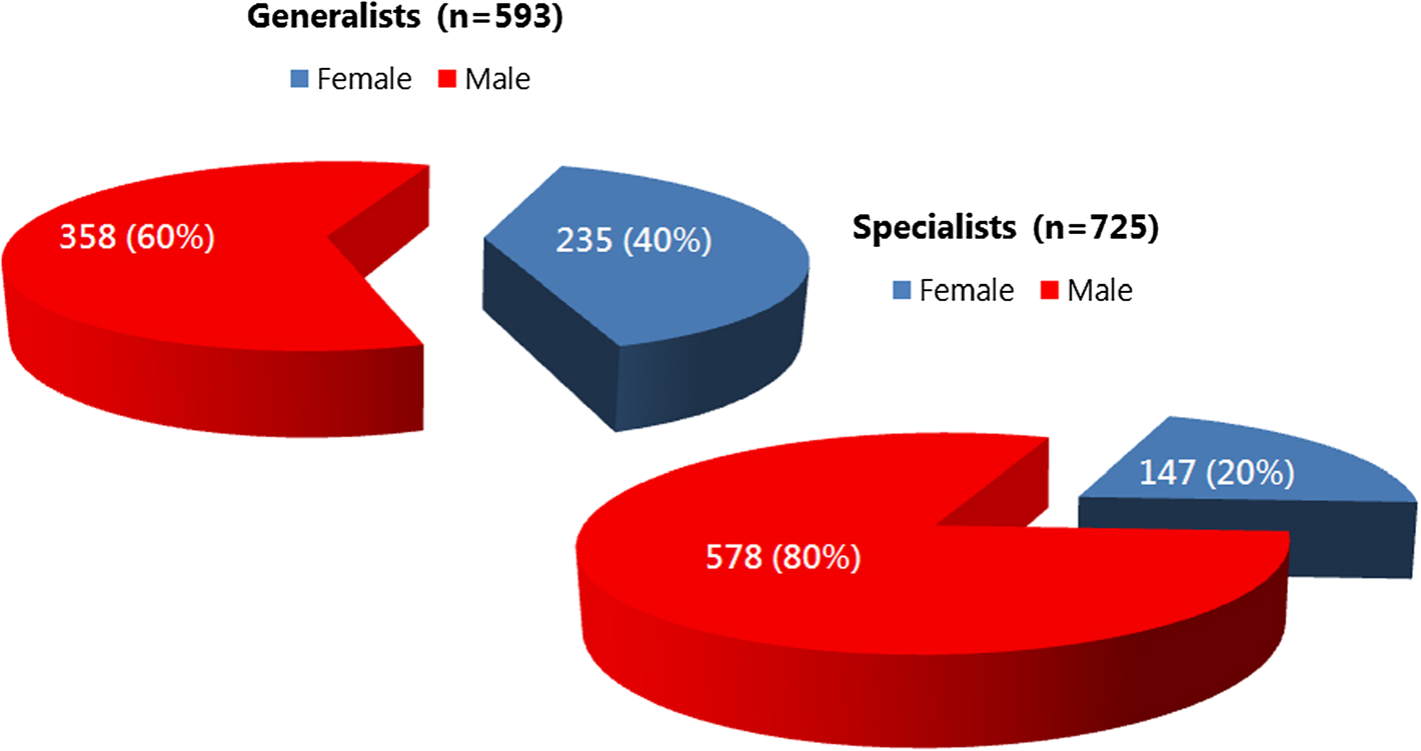
**Percentage of men and women in the medical practitioner cadre, Kenya 2011.**

A 2010 study of training institutions using a stratified random sample used focus group and sex-disaggregated administrative data to explore gender-related barriers in health pre-service education [12]. Analysis of the administrative data found distinct differences in the concentrations of female and male *students* in different occupational cadres. In Figure 1, some occupations - such as nursing, nutrition, community health work (CHW), and community health extension work (CHEW) - appear to be ‘female jobs’ whereas pharmacy appears to be a ‘male job’. Figure 2 highlights heavier concentrations of men in five of eight *faculty* categories in nursing-only educational institutions. Later, gender reports were generated from national HRIS data to explore the gender concentration within the medical cadre (Figure 3). All these results indicate different dimensions of horizontal gender segregation.

A noted labour economist observed that occupational segregation points to discrimination and limited opportunities because ‘When large segments of the labour force are in essence restricted from entering many occupations, freedom of choice is missing’ [33]. The Kenya findings suggest a lack of freedom of choice for education and employment that is associated with occupational segregation. In response, the Kenyan Ministry of Health integrated HRIS gender reports in a training module in 2013 to raise awareness of gender inequality in human resources management (HRM).

#### *Uganda*

**Figure 4:**
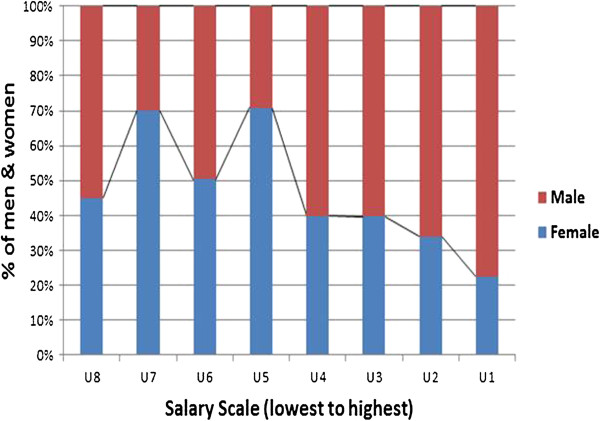
**Percentage of men and women by pay grade, public health sector in 12 sites, Uganda 2012 (N = 6,450).**

**Figure 5:**
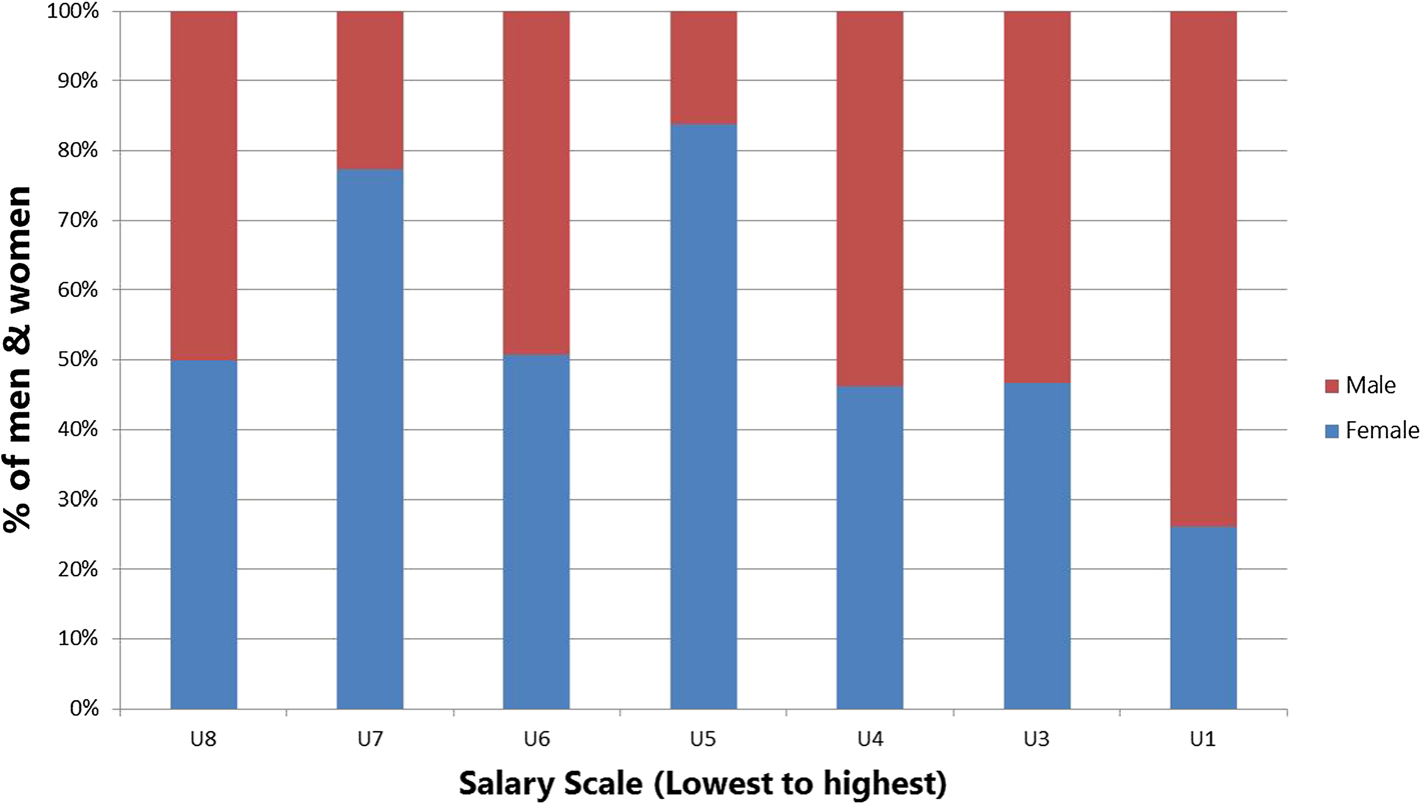
**Percentage of men and women by pay grade, Mulago National Referral Hospital, Uganda 2012 (N = 2,186).**

**Figure 6:**
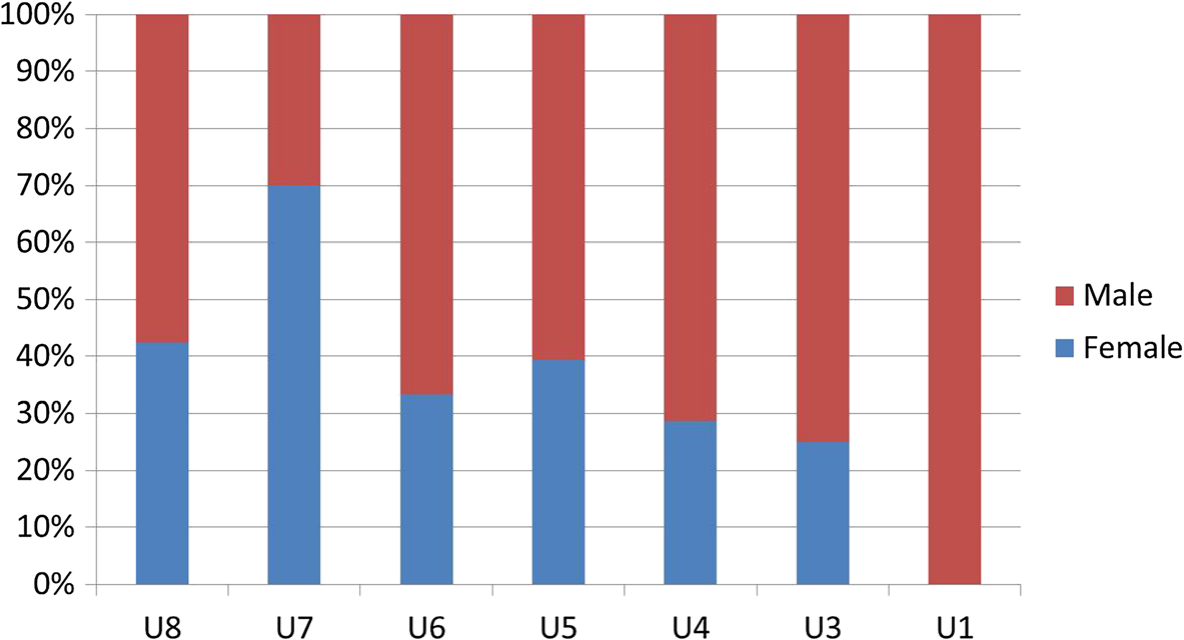
**Percentage of women and men by pay grade, Mubende Referral Hospital, Uganda 2012 (N = 183).**

**Figure 7:**
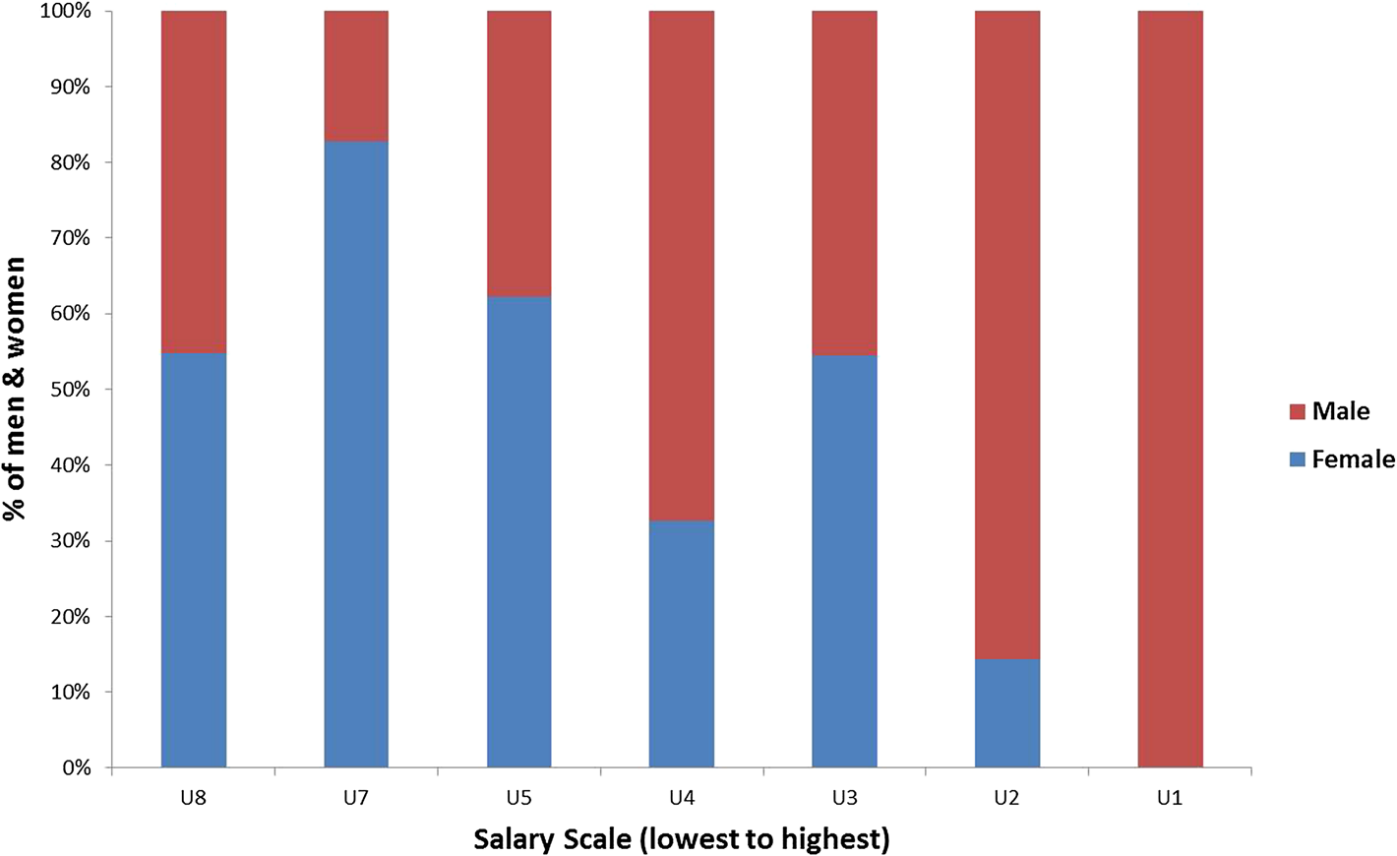
**Percentage of men and women by pay grade, Moroto Regional Referral Hospital, Uganda 2012 (N = 161).**

Researchers supplemented focus group, interview, and document review data collected in 12 programme sites with analyses of public sector position grade and salary data from these sites in the national HRIS. The position grade categories included: U1 (senior management); U2-U3 (middle management); U4-U5 (graduate and diploma levels); and U6-U8 (lower-level cadres). The HRIS gender reports revealed concentrations of male and female health workers by pay scale level (as in Figure 4, which shows vertical segregation and less female representation at the higher position levels aggregately from 12 sites). Figure 5, Figure 6, and Figure 7 illustrate a similar higher concentration of male workers in the senior management positions (U1-U2) in three referral hospitals. These figures show an important multi-site trend in referral hospitals illustrating a systemic problem. The value of the pay grade data in Figures 4, 5, 6, and 7 is that they clearly show the link between vertical gender segregation and gender wage gaps.

It should be noted that the Uganda findings come from health facilities in project districts purposively selected to capture a range of health workplace characteristics (for example, urban/rural, types and levels of facilities, nearness/distance from the capital). They cannot, therefore, be called representative. What is interesting, however, is that these public health sector results generally mirror the patterns of vertical occupational segregation found in Uganda’s larger civil service sector [46], where men predominate in senior and middle management (U1 and U2-U3, presumably the higher-paying jobs). Based on the study, the Uganda Ministry of Health has begun disseminating results and guidelines for gender mainstreaming into human resources management at decentralized levels to raise awareness of these issues with district health managers.

### Lessons learned and implications for action

What underlies the gender-related inequalities in position, freedom of choice, and opportunity in the health workforce? To what extent do these inequalities reflect discrimination and contribute to problems in health workforce recruitment, distribution, and retention? Although the answers to these questions are in the early stages, it is clear that HRH leaders must consider gender discrimination and inequality as part of their health systems governance functions.

HRH leaders can draw a number of lessons from the research evidence from systematic [45] and country-level studies. First, taken together, the evidence suggests that gender is indeed a key factor in the health workforce, operating in the professional education and employment systems in which health workers are recruited, trained, hired, remunerated, promoted, and retained - or lost. Second, there is evidence of a constellation of gender discrimination effects that are systemic, that is, not limited to one site in a system or one system. Third, the types of workplace discrimination documented in other sectors appear to be at work in the health sector. Fourth, the existing evidence warrants making gender inequality and gender discrimination an HRH research, policy, and management priority. Fifth, HRH leaders and managers should exploit data from multiple sources and perspectives to more fully understand gender dynamics and trends in the health workforce.

As Table 4 indicates, there is a need for a unified conceptual framework for gender inequality and discrimination in the health workforce and routine information to increase understanding of prevalent types, forms, and health systems consequences. Future HRH research should systematically explore the extent of occupational segregation and the gender wage gap, along with other forms of discrimination, perceptions of equal opportunity, and prevailing stereotypes of men and women in the health workforce in relation to recruitment, job assignment, promotion, geographical distribution, and retention. As mentioned earlier, discrimination can be difficult to measure due to differing perceptions, measurement approaches, unwillingness to publicly acknowledge or report it, and lack of information substantiating it. This suggests that research should routinely employ mixed qualitative and quantitative methods to address discrimination from several angles and better triangulate data. Where focus group discussions are used, the group composition should facilitate open and frank discussion (for example, members should be homogeneous for characteristics such as sex and hierarchical rung). Where survey methods are used, large-scale random sampling would enable HRH decision-makers to begin to grasp the extent and magnitude of the gender discrimination and inequality operating in their health workforces.

**Table 4:** Implications for action

	
**GLOBAL ACTIONS**	
**Develop a conceptual framework:**	A unified conceptual framework for gender in the health workforce would span pre-service and continuing education and employment systems and include a taxonomy with significant gender inequalities as they operate in the health workforce, including gender discrimination and inequalities defined in measurable terms and workforce and health systems consequences.
	Possible consequences: clogged health worker educational pipelines, recruitment bottlenecks, attrition, lower productivity, worker maldistribution.
**Produce research guidance:**	A community of gender and HRH research practice similar to the *Joint Programme on Workplace Violence in the Health Sector*[47] should produce research guidance based on the conceptual framework, identifying a gender and HRH research agenda and developing guidelines for systematic research.
	Practice community: representatives from UN Women, World Health Organization, International Labour Office, Global Health Workforce Alliance, International Council of Nurses, Public Services International, and nongovernmental organizations (NGOs) specializing in HRH and health systems strengthening.
**Improve global HRH governance in health systems strengthening efforts:**	Bring international human/labour rights and employment law discourse into HRH discourse, develop sample HRH policies to reflect this, and integrate human/labour rights and gender equality principles into global consensus documents. (Note: gender equality in the workforce will require cooperation between governments, workers’ unions, professional associations, and NGOs.)
	Consensus documents: declaration following the next global HRH forum; WHO Global Code of Practice on the International Recruitment of Health Personnel; guidelines for HRH assessments and observatories.
**COUNTRY ACTIONS**	
**Reform national HRH leadership and governance:**	Apply the protections available to workers in international human rights conventions, national constitutions, equal opportunity policies and laws, and labour codes to national HRH policies and HRM practice standards.
	Examples: adapt affirmative action policies to health worker recruitment or promotion initiatives; raise HRH stakeholders’ awareness of gender in the workforce through training; strengthen HRH leaders’ capacity to use HRIS gender reports to identify gender trends in the workforce as the basis of HRH strategies; and conduct country-specific gender and HRH research.
**INSTITUTIONAL ACTIONS**	
**Improve institutional HRH governance by equalizing opportunity and promoting gender equality in health education settings and workplaces:**	Anticipate health workers’ lifecycle needs, recognizing that sociocultural factors call for vigilance to assure equal opportunities, nondiscrimination, and gender equality in the workforce. This entails developing workplace policies, allocating resources, and restructuring education and work settings to integrate family and work and reflect the value of caregiving for women and men.
	Examples: prohibit workplace discrimination through nondiscrimination and equal opportunity policies. Make it easier to integrate work and family life, by: including paid maternity, paternity, and parental leave; offering part and flexible-time options, job sharing and access to child care in incentives packages; revising any workplace policy or practice that directly or indirectly privileges unmarried or childless workers in hiring, pay, promotion, and so on, or that penalizes female health workers because of marriage, pregnancy, motherhood, and family caregiving status.

Finally, while we need more systematic and rigorous evidence and better-developed understandings, HRH leaders and managers can now strive for greater clarity. For example, the Third Global HRH Forum’s *Political Declaration on Human Resources for Health* states that ‘gender imbalances’ remain a matter of major concern [48], but it is not clear what ‘imbalances’ means. However, when the Declaration commits to ‘promote equal opportunities in education, development, management and career advancement for all health workers, with no form of discrimination based on gender, race, ethnicity or any other basis’ - we are witnessing a clear HRH governance milestone. One hopes that a Fourth Global Forum will not only commit to ending gender discrimination but to actively promoting gender equality in the workforce through improved global HRH governance (see Table 4).

Good HRH governance starts with the recognition that the health workforce is diverse and acknowledges the fact that diversity often entails gendered disadvantage in accessing opportunities for education and occupation. It requires a comprehensive human rights-based approach that puts women at the center of efforts to hold governments and employers accountable for implementing international and national standards that guarantee women’s civil, social, political, and labour rights [49]. In the health sector, this means bringing international human/labour rights and employment law discourse into HRH discourse, changing discriminatory laws and policies, and developing HRH policies that promote and protect the rights of the health workforce which, in many countries, may be over 75% female [9]. Changing workforce governance at all levels is necessary because gender inequality and discrimination are human rights issues with practical workforce consequences (see Table 4). These practical consequences will be difficult to manage effectively if HRH practitioners continue to see action as a purely technical fix. However, to the extent that they more accurately identify the issues, apply a human rights perspective, draw lessons from employment law in other sectors, and broaden the range of HRH solutions available, there is good reason to expect that these steps will improve the pipeline, recruitment, distribution, and retention of health workers.

## Conclusions

Gender equality should be an HRH research, leadership, and governance priority. As a priority, the aim should be to strengthen health pre-service and continuing professional education and employment systems to achieve better health systems outcomes, including better health coverage. There are a number of specific actions that can be carried out at the global, country, and institution levels to address gender discrimination and inequality in the health workforce (Table 4), some of which have already been described. Implementing any of the suggested actions will likely move us closer to the robust health workforces needed to respond to today’s critical health care needs.

## Abbreviations

CHEW: community health extension work; CHW: community health work; HR: human resource; HRH: human resources for health; HRIS: human resources information system; HRM: human resources management; NGOs: nongovernmental organizations; USD: United States dollars.

## Competing interests

The author declares that she has no competing interests.
